# The role of metformin in treatment of weight gain associated with atypical antipsychotic treatment in children and adolescents: A systematic review and meta-analysis of randomized controlled trials

**DOI:** 10.3389/fpsyt.2022.933570

**Published:** 2022-11-15

**Authors:** Zeeshan Mansuri, Ramkrishna Makani, Chintan Trivedi, Mahwish Adnan, Ramu Vadukapuram, John Rafael, Ashutosh Lodhi, Abhishek Reddy

**Affiliations:** ^1^Department of Psychiatry, Boston Children’s Hospital and Harvard Medical School, Boston, MA, United States; ^2^Department of Psychiatry, AtlantiCare Health System, Egg Harbor Township, NJ, United States; ^3^Department of Psychiatry, Texas Tech University Health Sciences Center at Permian Basin, Odessa, TX, United States; ^4^The University of Texas Rio Grande Valley, Harlingen, TX, United States; ^5^MS4, Texas Tech University Health Sciences Center at Permian Basin, Odessa, TX, United States; ^6^Providence Medical Group, Beaverton, OR, United States; ^7^Department of Psychiatry, Virginia Tech Carilion School of Medicine, Roanoke, VA, United States

**Keywords:** metformin, weight gain, antipsychotics, children, adolescents

## Abstract

**Introduction:**

Second-generation antipsychotics are associated with significant weight gain. The aim of this systematic review and meta-analysis was to determine the efficacy and safety of metformin for the treatment of weight gain in children and young adults treated with second-generation antipsychotics.

**Methods:**

We followed PRISMA guidelines to evaluated studies published before March 2020 in Medline, Google Scholar, PubMed, Cochrane library database, annual scientific sessions of the American Psychiatric Association, American Academy of Child and Adolescent, Psychiatry, and American Society of Clinical Psychopharmacology. Studies included compared metformin with the placebo for management of weight gain in children and adolescents taking atypical antipsychotics. Non-randomized studies, animal experiment studies, editorials, and review studies were excluded. Multiple parameters, including change in anthropometric-biochemical parameters, drug discontinuation rate, and side effects among the groups were assessed. The random-effects method was used for meta-analysis.

**Results:**

Four studies with were included in the final analysis (213 patients; metformin: 106; control: 107). After pooled analysis, 12–16 weeks of metformin therapy was associated with a significant reduction in weight [(mean difference (MD): −4.53 lbs, confidence interval (CI): −6.19 to −2.87, *p*-value < 0.001)], and BMI z score [MD, −0.09, CI: −0.16, −0.03, *p*-value: 0.004] compared to control. Metformin was also associated with a significant reduction in insulin resistance [MD: −1.38, CI: −2.26 to −0.51, *p*-value: 0.002]. There were higher odds of nausea-vomiting [OR: 4.07, CI: 1.32–12.54, *p*-value: 0.02] and diarrhea [OR: 2.93, CI: 1.50–5.71, *p*-value: 0.002] in the metformin group. However, there was no difference in drug discontinuation rate [OR: 1.45, CI: 0.41–5.06, *p*-value: 0.56].

**Conclusion:**

Metformin may prove beneficial in the treatment of weight gain in children treated with second-generation antipsychotics. The pooled treatment effect showed a significant reduction in BMI Z-score and weight in just 12–16 weeks. The limitations include small sample size, variation in metformin dose, and duration of treatment. This meta-analysis should be interpreted as promising, and further larger studies are warranted before drawing a conclusion.

## Background

Antipsychotics are used in the child and adolescent patient population to treat psychosis, treatment-resistant depression, bipolar disorder, ADHD, and anger/irritability associated with autism in children, as well as used off-label to treat ailments ranging from conduct disorder to hyperkinetic disorder, among others (1–4). According to a study, 14% of 11-year old children have experienced psychotic symptoms, which are associated with a 5 to 16 times increase rate of psychotic illness in early adulthood (1). In another published study in the adolescent population, 9–14% reported experiencing psychotic symptoms (5, 6). The symptoms of psychosis in childhood and adolescence are concerning as they are more likely to be associated with schizophrenia, bipolar disorder, autism spectrum disorders, attention-deficit/hyperactivity disorder, delirium, post-traumatic stress disorder, schizoaffective disorder, and major depressive disorder (2). According to the American Psychiatric Association (APA) guidelines, antipsychotic medication is a recommended treatment in individuals with psychosis and other indications listed above. It should be administered in the acute phase as well as throughout the maintenance and recovery phase.

Antipsychotics are highly effective and well-tolerated in the treatment of psychosis (7). First-generation antipsychotics are effective but are associated with tardive dyskinesia and extrapyramidal side effects (8). Hence, second-generation antipsychotics are preferred in children with psychosis, mood disorders, impulse control disorders, and disruptive behaviors in the context of autism spectrum disorders. Second-generation antipsychotics have shown good efficacy; however, they are often associated with significant weight gain, which becomes a significant limiting factor (9–13). Weight gain in early childhood is alarming as childhood obesity is already a growing epidemic in recent years (14, 15). Obese children are more prone to cardiovascular, metabolic, and psychiatric disorders. (16–22). Hence, it is critical to have a timely intervention for weight gain, especially if it is drug-induced.

Metformin is a biguanide drug that is widely used to treat type 2 diabetes and works by increasing insulin sensitivity, decreasing glucose production in the liver, and decreasing intestinal glucose absorption. Metformin is also known to increase peripheral utilization of glucose and suppress appetite (23). It may contribute to weight reduction by reducing insulin resistance, decreased production and increased peripheral utilization of glucose (24). In recent years, several studies have shown the benefits of metformin in patients with antipsychotic-induced weight gain and metabolic abnormalities, where metformin inclusion is an effective, safe, and reasonable choice (24–27). Despite the number of publications on this topic in the adult population, very few studies were conducted in children and adolescents. One of Morrison et al.’s earliest single group open label studies on off-label use of metformin showed that significant weight loss could occur in pediatric patients on psychotropic drugs treated with metformin (28). Subsequently, three randomized clinical trials with small sample size studies were published, which showed promising results of metformin in antipsychotic associated weight gain (29–31). A recent randomized clinical trial (32) “metformin” was compared with “no metformin” as well as with the “lower risk antipsychotic switch” group. The metformin group showed excellent results by improving not only anthropometric parameters but also psychotic symptoms.

We perform a systematic review and meta-analysis of current literature to determine the efficacy and safety of metformin for the treatment of weight gain in children and young adults treated with second-generation antipsychotics.

## Materials and methods

### Search strategy and study selection

**FIGURE 1:**
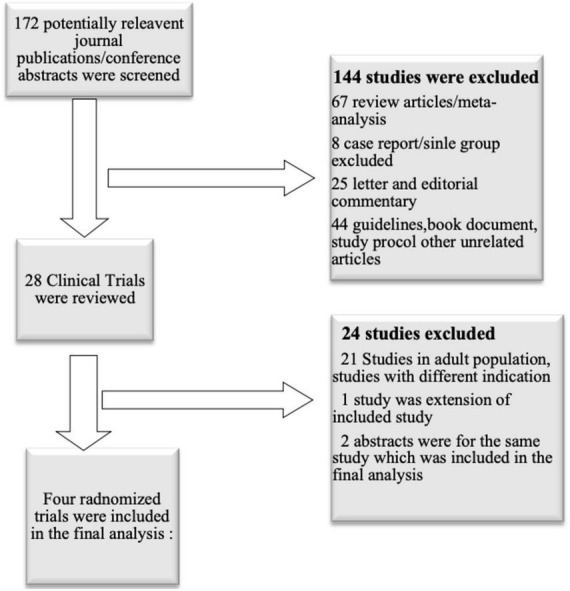
Selection process of studies included in the systematic review.

We evaluated all the relevant studies published before March 2020. We included all the studies performed in children and adolescents, where metformin was used and compared with the placebo for the weight gain management in patients taking atypical antipsychotics. The studies were searched using MeSH (Medical Subject Headings) term “metformin”, “overweight”, “child”, “adolescent”, “antipsychotic agents”, “Increased body weight” (Figure 1). Studies were searched from Medline, Google Scholar, PubMed, Cochrane library database, annual scientific sessions of the American Psychiatric Association, American Academy of Child and Adolescent, Psychiatry, and American Society of Clinical Psychopharmacology.

All non-randomized, animal experiment studies, editorial, and review studies were excluded. Selection of Studies and Data Extraction was conducted in two separate stages. In the first stage, two researchers (C.T., MA) independently screened all non-duplicate references initially retrieved as potentially pertinent and excluded those not relevant based on title or abstract. A final list was agreed on, with discrepancies resolved by consensus between the two authors. In the second stage, full-text versions of the articles passing initial screening were downloaded and independently assessed for eligibility by the researchers. Data were collected from all the studies for baseline characteristics, change in anthropometric-biochemical parameters, drug discontinuation rate, and side effects of both the groups.

### Statistical analysis

To provide an overall estimate of the effect of metformin therapy on the treatment of weight gain in children and young adults treated with second-generation antipsychotics, we performed a meta-analysis by including the four randomized clinical studies (29–32). Weight data were converted to lbs from kg for three studies, and in one of the studies, the standard error was converted into standard deviation. The presence of heterogeneity among these studies was evaluated with the Cochrane Q χ^2^ test. The inconsistency was assessed with the I2 test that describes the percentage of the variability in effect estimates that is due to heterogeneity. Publication bias was assessed by visual inspection of the funnel plot of precision. The statistical level of significance for the summary treatment effect estimate was analyzed in a random effect model by inverse variance and Mantel- Haenszel method (33). Overall, a *p*-value of less than 0.05 was considered statistically significant except for heterogeneity and publication bias testing, where a two-tailed *p*-value of less than 0.1 is considered statistically significant. The meta-analysis was performed by the review manager 5.3 software (34).

## Results

### Study characteristics

A total of 172 journal publications or conference abstracts were screened. Out of those, 144 studies excluded because they were review articles, meta-analysis, study protocol publications, book chapters, editorial, or commentary on a published article. The remaining 28 clinical trials were reviewed, and after evaluating the study design, and relevant age group, seven studies were evaluated. Out of seven, one was an extension of the included study, and two were abstracts of included study. Finally, four randomized clinical trials (two single-center and two multicenter studies) conducted in children or young adults were included in the final analysis (26–29) (Figure 1).

Tables 1, 2 summarize the study and population characteristics, respectively. All the patients were only on single second-generation antipsychotic medications. The mean age was between 11 and 13 years in all the studies, and almost two-thirds of the patients were male. In the study by Arman et al., the average BMI was 17; however, in other studies, the average BMI was between 26 and 30. Similarly, in the study by Arman et al., the average weight was only 78 lbs; however, it was between 150–170 lbs in other studies. Only two studies provided baseline information on fasting glucose, insulin, and insulin resistance (Table 1).

**TABLE 1:** Characteristics of the included studies in the meta-analysis.

Study characteristics	Klein et al. (29)	Arman et al. (30)	Anagnostu et al. (40)	Correll et al. (32)
Number of patients included in the study	38	32	60	96
Design	Single center, placebo controlled randomized trial	Single center, placebo controlled randomized trial	Multi center, placebo controlled randomized block design with block size of two and four	Multi center, randomized, unmasked parallel group clinical trial
Follow up at clinic	4, 8, 12, and 16 weeks	4 and 12 weeks	2, 4, 8, 12, and 16 weeks after treatment initiation	1, 2, 4, 6, 8, 12, 16, 20 and 24 weeks
Psychiatric diagnoses	Bipolar disorder, Attentional disorders, Schizophrenia, Oppositional defiant Disorder, Autism and Asperger’s syndrome, Tourette’s syndrome, Schizoaffective disorder, and Depression	Schizophrenia	Autistic disorder, Asperger disorder, or pervasive developmental disorder not otherwise specified	Schizophrenia spectrum disorder, Bipolar spectrum disorder, or Major depression with psychotic features, ADHD, Autism spectrum disorder, Anxiety disorders, Oppositional defiant disorder/conduct disorder
Antipsychotic medications*	Risperidone, Olanzapine, Quetiapine	Risperidone	Risperidone, Aripiprazole	Risperidone, Aripiprazole
Intervention	500 mg metformin or an identical-appearing placebo at their evening meal for 1 week, In second week, second dose was added before breakfast. After second week, the dose was increased to 850 mg given with these meals for an additional 14 weeks.	500 mg Metformin in the morning or identical placebo for 1st week After that, two tablets, one in the morning, one at night	Metformin or placebo titrated up to 500 mg twice daily for children 6–9 years and 850 mg twice daily for those 10–17 years	For <50 kg, the metformin started with 250 mg, which was increased by 250 mg after 1 week, and subsequently by 250 mg increase on a weekly basis, until 500 mg twice daily was reached. For 50–70 kg, max dose was 500 mg in the morning and 1,000 mg in the evening. For >70 kg, the metformin started with 500 mg taken with dinner, which was increased by 500 mg after 1 week, and subsequently by 500 mg steps on a weekly basis until 1,000 mg twice daily was reached
Diet and nutritional counseling	Yes	No	Yes	Yes
Primary end Point	Anthropometric measures (weight, BMI) change from baseline at week 4, week 8, week 12, week 16	Weight and BMI change at 4 weeks and 12 weeks	Weight and BMI Z score change at 16 weeks	BMI z-score change at week 12 and week 24

*Only antipsychotics included if at least 10% of them were taking it.

**TABLE 2:** Baseline characteristics of study patients.

	Klein et al. (29)		Arman et al. (30)		Anagnostu et al. (40)		Correll et al. (32)	
Variables	Metformin (*n* = 18)	Placebo (*n* = 20)	Metformin (*n* = 16)	Placebo (*n* = 16)	Metformin (*n* = 28)	Placebo (*n* = 32)	Metformin (*n* = 49)	Placebo (*n* = 47)
Age, years	12.9 ± 2.4	13.3 ± 2.4	11.3 ± 2.5	8.9 ± 4.3	12.9 ± 2.9	12.7 ± 2.6	13.4 ± 3.2	13.4 ± 3.2
Male	9 (50)	12 (60)	11 (69)	10 (63)	21 (75)	24 (75)	31 (63)	30 (64)
BMI	26.7 ± 5.4	28.7 ± 7.0	17.4 ± 2.8	17.0 ± 4.4	29.8 ± 6.1	29.8 ± 5.4	–	–
Weight, kg	67.2 ± 22.6	74.3 ± 28.1	35.2 ± 12.9	29.8 ± 20.0	77.5 ± 25.4	76.4 ± 23.0	–	–
HOMA-IR	5.08 ± 6.9	4.81 ± 3.8	–	–	3.45 ± 1.9	4.56 ± 3.3	–	–
Insulin level (μU/ml)	20.4 ± 19.7	21.1 ± 14.6	–	–	16.6 ± 10.1	21.6 ± 14.8	–	–
Glucose level (mg/dl)	87.8 ± 14.2	88 ± 12.9	–	–	87.1 ± 9.3	85.9 ± 9.9	–	–

Values are reported as mean ± SD or *n* (%). Ω in the efficacy analysis 15 patients included in each group as eight patients dropped out.

Klein et al. assessed metformin treatment of weight gain associated with the initiation of atypical antipsychotic therapy in a 16-week double-blind study performed in patients of 10–17 years of age. Only patients who gained >10% of their pre-drug weights in less than a year of treatment with atypical antipsychotic agents were included. All patients were on a stable dose of antipsychotics for at least 3 months. Patients were followed up at weeks 4, 8, 12, and 16 for anthropometric measurements and interviewed for side effects. At each visit, blood samples were obtained for lactic acid, sodium, potassium, BUN, creatinine, bicarbonate, liver enzymes, and pregnancy testing. Also, blood samples were obtained for insulin levels, insulin resistance, and fasting glucose blood level at weeks 8 and 16. All patients received nutritional counseling at weeks 4, 8, and 12.

Arman et al. evaluated the effect of metformin vs. placebo on treatment on the risperidone induced weight gain in patients with Schizophrenia or Schizoaffective disorder. It was a 12-week double-blind clinical trial where all patients were <20 years of age and were taking risperidone and were antipsychotic naïve. A total of 49 patients were recruited; however, 17 of 49 were excluded due to side effects or not able to complete the medication protocol. Patients were followed up weekly for side effects monitoring by phone follow up. In addition, data on anthropometric (BMI, weight, height) and biochemical parameters (fasting blood glucose, CBC, creatinine, prolactin level, liver function) were collected at week four and week 12. Patients in this study had very low BMI (mean: 17 kg/m^2^) and almost half the weight on an average compared to other studies.

Anagnostu et al. compared the efficacy of metformin to placebo for weight gain associated with atypical antipsychotic medications in children and adolescents with an autism spectrum disorder. It was a 16-week multicenter, double-blind study where 61 patients were randomized, but one patient was excluded, as he could not take liquid medication. All patients were on a stable dose of an atypical antipsychotic for at least 30 days. Patients were included if there was a documentation of 7% or more increase in BMI since starting an atypical antipsychotic within the past 1 year, or if the BMI ≥ 85th percentile, and >5% bodyweight increase per year since starting the medication. Patients with the previous usage of metformin were excluded. Height and weight were checked at baseline, weeks 2, 4, 8, 12, and 16 after starting the treatment, and clinical laboratory tests were performed at baseline and 16 weeks. All patients received brief counseling regarding diet and exercise. The laboratory tests were performed at baseline and 16 weeks of follow-up.

In a recently published study by Correll et al., overweight or obese youth with severe mental illness on antipsychotics were evaluated in three groups: metformin add-on vs. antipsychotic switch vs. continued antipsychotic treatment plus healthy lifestyle education. Patients were 8–19 years old, and they were treated with second-generation antipsychotics with a stable dose for at least 30 days. Patients were included if they were clinically stable on the current treatment regimen for at least 30 days, having BMI greater or equal to 85th percentile for age-gender, and substantial weight gain of at least 10% of baseline weight. It was a 24-week study that involved ten in-person visits at baseline, weeks 1, 2, 4, 6, 8, 12, 16, 20, 24, and 6 phone sessions at weeks 3, 5, 7, 9, 10, 11 weeks. Also, in this study, psychiatric symptomatology was assessed at follow-up. All the laboratory values were obtained at baseline, 12 weeks and 24 weeks.

### Efficacy outcome

**FIGURE 2:**
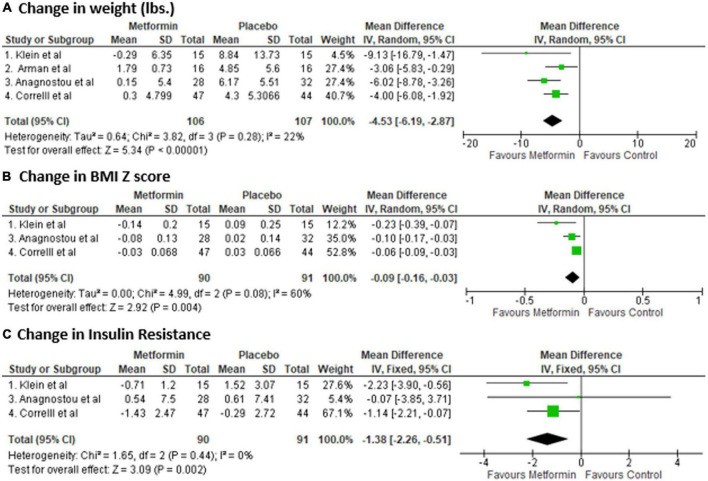
Forest plot showing the Mean Difference (MD) and Standard Deviation (SD) of **(A)** change in weight, **(B)** BMI Z score, and **(C)** insulin resistance in Metformin and Placebo group of each study. Square boxes denote MD; horizontal lines represent 95% confidence interval (CI) (random effect model was used to calculate pooled estimate). The vertical solid line represents no difference between Metformin and control. Pooled MD (95% CI) between Metformin and Placebo are represented by diamond shape.

A total of 213 patients were included in the final analysis; 106 patients in metformin and 107 patients in the control group. After pooled analysis, metformin group was associated with a significant reduction in weight from baseline compared to control [Mean difference (MD): −4.53 lbs, 95% confidence interval (CI) (−6.19, −2.87), *p*-value: <0.001] (Figure 2). Since, body weight in the study by Arman et al. was a outlier, we performed pooled analysis again after excluding the Arman et al. study, and found even stronger effect of metformin on body weight [Mean difference (MD): −5.09 lbs, 95% confidence interval (CI) (−7.07, −3.11), *p*-value: <0.001]. Similarly, BMI z score reduced (*n* = 3 studies, as lack of data in Arman et al. study) significantly in metformin group [MD, −0.09, 95% CI: (−0.16, −0.03), *p*-value: <0.001]. There was no heterogeneity in weight change analysis [I2: 22%], however, in BMI-z score change analysis there was a moderate heterogeneity [I2: 60%]. In addition, metformin was associated with reduction in insulin resistance [MD: −1.38, 95% CI: −2.26 to −0.51, *p*-value: 0.002, I2: 0%]. Data on biochemical parameters were available for only two studies [28–29], and it showed beneficial effect for all parameters; however, it was non-significant change [fasting glucose (MD −4.93, *p*-value: 0.25), insulin (MD −4.73, *p*-value: 0.51), HbA1C (MD −0.07, *p*-value: 0.09), total cholesterol (MD −3.75, *p*-value: 0.54), LDL (MD −5.88, *p*-value: 0.06), and HDL (MD + 1.68, *p*-value: 0.37)].

### Safety outcome

**FIGURE 3:**
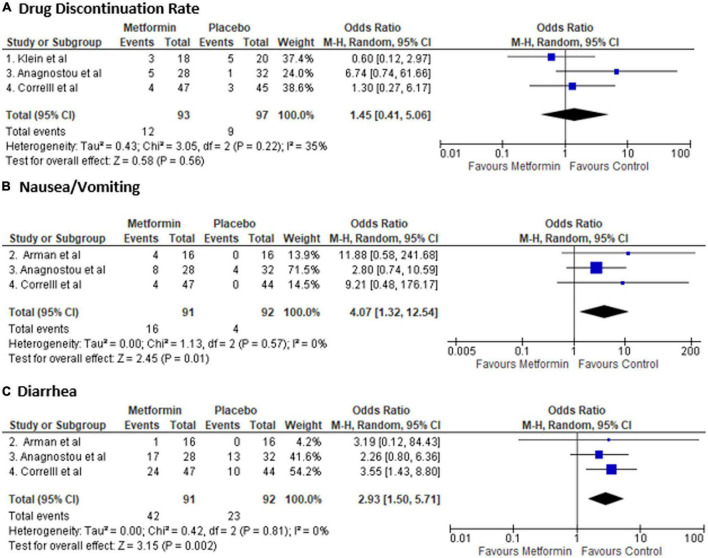
Forest plot showing Odds Ratio (OR) and 95% confidence interval (CI) for rate of **(A)** drug discontinuation **(B)** nausea and vomiting, and **(C)** diarrhea in each study and overall Odds Ratio. Square boxes denote OR; horizontal lines represent 95% CI (random effect model was used to calculate pooled estimate). The vertical solid line represents no difference between Metformin and control. Pooled OR (95% CI) between Metformin and Placebo are represented by diamond shape.

There was no difference in drug discontinuation rate [Odds ratio: 1.45 (0.41–5.06), *p*-value: 0.14]; however, metformin was associated with higher number of nausea and vomiting [OR: 4.07 (1.32–12.54), *p*-value: 0.02] and diarrhea [OR: 2.93 (1.50–5.71), *p*-value: 0.002] (Figure 3). There was no heterogeneity in safety outcome analysis [I2: 0%]. In the study by (29), side effects were similar. However, there was no data on the type of side effects that is why it was not included in the analysis. Also, in a study by (30), three patients in the metformin group had nausea and vomiting, and one in each group had diarrhea. However, they were excluded from the study before randomization, so they were not included in the analysis.

## Discussion

This is the first meta-analysis focusing on the efficacy as well as the safety of metformin treatment for weight gain in children and adolescents undergoing treatment with second-generation antipsychotic medications. Second-generation antipsychotics are very effective medications, but the side effect of weight gain is a significant concern (9–13). There was a systematic review and meta-analysis on this subject by Ellul et al. (35). However, in that analysis, efficacy was analyzed by calculating weight and BMI score change from three published and two non-published studies. In our study, we analyzed BMI z-score change since it is more relevant in growing children. We also analyzed weight change, insulin resistance change, drug discontinuation rate, and side effects like nausea, vomiting, and diarrhea. The finding from our study shows that metformin is associated with a significant reduction in weight compared to the placebo/control group.

According to the pooled analysis of randomized clinical trials, at 12–16 weeks after starting the treatment, metformin was associated with almost 5 lbs reduction in weight compared to the placebo. Moreover, it was associated with a significant reduction in BMI z-score and insulin resistance. There was more reduction in body weight and BMI z-score at 24 weeks of follow-up. Overall, patients in the metformin group had fewer problems with aggression/hostility; which imply that it also helped in symptom relief (32). There was an improvement in biochemical parameters such as cholesterol, glucose, LDL, HDL, and insulin level, but it was not statistically significant. Despite a statistically significant effect on body weight, the nearly 5 lbs reduction may not be clinically relevant given the relatively small sample size and the efficacy of lifestyle modifications, including diet and exercise, that may treat weight gain and have other positive effects. Moreover, metformin was associated with more adverse events with a nearly four times higher rate of nausea-vomiting and approximately three times higher diarrhea rate than placebo. However, the majority of the side effects were not serious adverse events. Patients showed good compliance with the treatment with no significant difference in the drug discontinuation rates between the groups. The majority of the side effects were gastrointestinal side effects, which can easily be reduced by using extended-release formulation and taking it with a large meal (36).

Recent studies have shown that schizophrenia may itself contribute to metabolic syndrome. Lang et al. conducted a study on 430 first-episode drug-naïve schizophrenia patients. Authors reported that the metabolic syndrome co-existed in the early stages of schizophrenia in the absence of antipsychotic treatment (37). Also, there might be a differences in prevalence of weight gain depending on the observation period of a study, stage of schizophrenia at which a patient presents, and the history of antipsychotics used, in combinations and consecutions (38, 39). Thus, it is imperative to assess the evidence for the use of metformin in addressing obesity in children and adolescents with schizophrenia, especially due to medication use. Since second generation antipsychotics are widely prescribed (38), it is critical to study the efficacy and safety of metformin for management of weight gain in patients with schizophrenia on second generation antipsychotics. Greater number and high powered randomized clinical trials, especially in the age group below 18, are required to assess role of metformin in the management metabolic syndrome in children and adolescents diagnosed with schizophrenia on second generation antipsychotics.

Despite promising results, our study has several limitations. First, the overall sample size was small despite four studies. Second, the dose and duration of metformin were different in each study. Third, inclusion criteria for weight gain before the study were different in each study and in the study by Arman et al. (30), where it was more like a preventative strategy for weight gain instead of treatment. That is why those patients’ weight and BMI were significantly less compared to the other three studies. Fourth, in one study (32), a placebo was not administered in the control group, and in two studies (29, 30), fixed metformin dose was given instead of titrated dose for all age groups. Fifth, drug discontinuation, specifically due to metformin related adverse events, was not provided among two studies. Finally, we analyzed the follow-up data at 12 weeks for two studies and at 16 weeks for two studies since there was a discrepancy in the follow-up data points. Despite the limitations, the role of metformin is promising in the treatment of weight gain in patients taking antipsychotics. It will help in designing future randomized clinical trials with a large patient population.

## Conclusion

Metformin was beneficial in treating weight gain in children and adolescents treated with second-generation antipsychotics in our meta-analysis of randomized controlled trials. Although the pooled treatment effect is significant with almost 5 lbs difference in just 12–16 weeks, a small number of studies and variation in metformin dose and duration of treatment in each study make our results promising but not conclusive and worthy of further investigation. The result should be interpreted as a positive hypothesis that can pave the way for a future clinical trial design that would address unanswered questions such as long-term outcome, change in psychiatric symptoms, metformin as a preventive strategy for weight gain, preferred dose, and length of therapy, and its specific use in the context of antipsychotic usage.

## Data availability statement

The original contributions presented in this study are included in the article/supplementary material, further inquiries can be directed to the corresponding author.

## Author contributions

ZM, CT, and AR contributed to the idea, writing, editing, and reviewing of the manuscript. RV and MA contributed to the writing, editing, and reviewing of the manuscript. RM, JR, and AL contributed to the reviewing of the manuscript. All authors contributed to the article and approved the submitted version.
